# Uric Acid Crystal Deposition Within a Ganglion Cyst: A Case Report

**DOI:** 10.7759/cureus.94625

**Published:** 2025-10-15

**Authors:** Kimia Targhi, Sadra Targhi, Melodie Mope

**Affiliations:** 1 Medicine, Florida State University College of Medicine, Tallahassee, USA; 2 Podiatric Medicine, Barry University School of Podiatric Medicine, Miami, USA; 3 Family Medicine, Florida State University College of Medicine, Tallahassee, USA

**Keywords:** exemestane, foot ganglion cyst, ganglion cyst aspiration, gout crystals, uric acid crystals

## Abstract

Ganglion cysts are soft tissue swellings occurring most commonly in the hand or wrist. They are typically filled with gelatinous material rich in hyaluronic acid and mucopolysaccharides. Although benign, variations in cyst content can provide important insight into underlying metabolic or microenvironmental processes. To date, uric acid crystal deposition within a ganglion cyst has not been reported. This case describes a 73-year-old woman with a history of breast cancer, treated with exemestane, who presented with a painful mass on the dorsum of the left midfoot. Physical examination revealed a fluctuant, soft tissue lesion without overlying erythema. Aspiration yielded yellow-tinged gelatinous fluid, which on histopathologic examination was consistent with a ganglion cyst. Under polarized light, needle-shaped crystals suggestive of monosodium urate were identified. The patient’s serum uric acid was at the upper limit of normal, and she reported arthralgias associated with exemestane therapy, but she had no clinical history of gout. This case represents an unusual presentation of a ganglion cyst containing uric acid crystals in the absence of gout or significant hyperuricemia. We propose that the mucopolysaccharide-rich microenvironment of the cyst may promote crystal precipitation independent of systemic metabolic disease. Exemestane-related tissue changes may also play a role, though further investigation is warranted. Recognition of this finding underscores the value of histopathologic evaluation in atypical cyst presentations and suggests potential links between local biochemical environments and crystal formation that merit future study.

## Introduction

Ganglion cysts are gelatinous, mucoid-filled synovial cysts. They are the most common soft-tissue masses found in the hand and wrist; however, they can also form in the knee and foot [1]. While benign, these lesions can cause discomfort, cosmetic concern, or functional limitation, depending on their size and location, and are a frequent reason for patients to seek medical attention. Management typically involves observation, aspiration, or surgical excision when symptomatic or recurrent [2].

Multiple explanations for the pathogenesis of ganglion cysts have been proposed, but no single theory has been universally accepted. Earlier theories suggested that they develop from synovial herniation through a joint capsule or from mucoid degeneration of connective tissue; however, the absence of a true synovial lining and the presence of a distinct cyst wall make these explanations less likely [3]. Current literature suggests that ganglion cysts originate from mesenchymal cells at the synovial-capsular junction, where ongoing microtrauma triggers fibroblasts to secrete hyaluronic acid [4]. The mucin produced may dissect through capsular and ligamentous structures, forming a cystic cavity that can communicate with the joint via a one-way valve mechanism [3]. The buildup of this hyaluronic acid-rich material results in the characteristic gelatinous contents of these cysts.

In contrast, uric acid crystal deposition typically occurs in articular or periarticular tissues as part of gout, an inflammatory process driven by supersaturation of monosodium urate in synovial fluid. These crystals commonly accumulate in joints such as the first metatarsophalangeal joint, where recurrent inflammation leads to tophus formation [5]. The relatively avascular, mucopolysaccharide-rich milieu of a ganglion cyst, however, lacks the inflammatory and vascular conditions usually required for crystal precipitation, making such deposition mechanistically unexpected.

The presence of uric acid crystals within a ganglion cyst is unusual and, to the best of our knowledge, has not previously been reported. The following is a report of a rare case of uric acid crystals within a ganglion cyst in a patient presenting with a painful lesion on the dorsum of her foot.

## Case presentation

**Figure 1:**
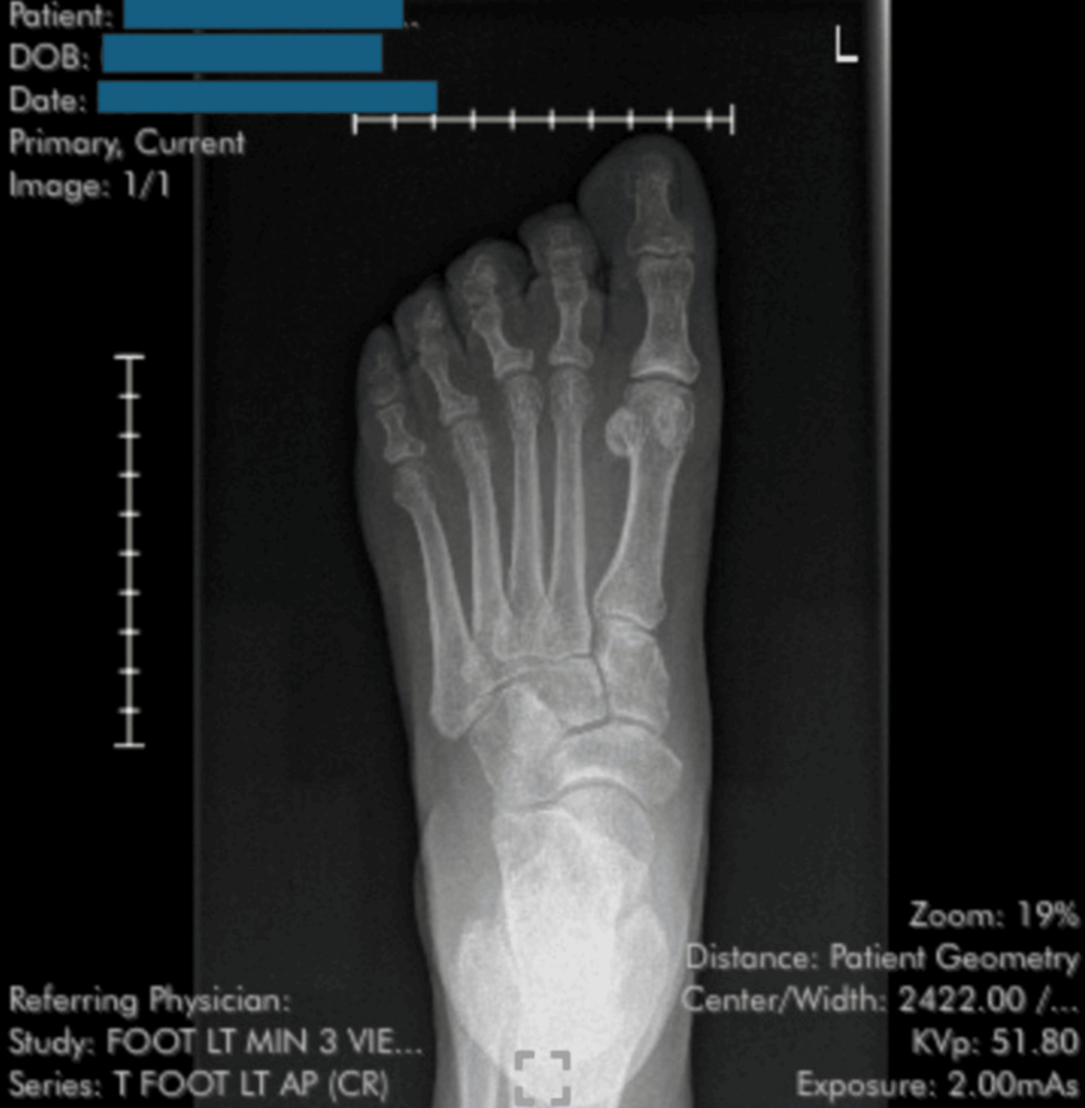
X-ray of the left foot showing no evidence of fracture or bony abnormality.

A 73-year-old woman with a medical history of breast cancer, treated with exemestane for the past five years, presented with a complaint of a painful lesion at the base of her left fourth metatarsal. She rated the pain as 4 out of 10 in intensity, with 0 representing no pain and 10 the worst pain imaginable. The pain was accompanied by localized tenderness on palpation and discomfort when wearing shoes. The lesion had been present for approximately two weeks before she sought evaluation at a local urgent care clinic, where plain radiographs of the foot were negative for fractures or other bony abnormalities (Figure 1). She was informed that a cystic lesion was suspected and was referred to podiatry for further management.

On physical examination, the lesion was a fluctuant, soft tissue mass overlying the dorsal-lateral aspect of the left midfoot at the base of the fourth metatarsal. The overlying skin was intact, without erythema or warmth, and the range of motion of the adjacent joints was preserved. However, direct palpation reproduced her pain. A clinical photograph was not obtained at the initial visit because the lesion appeared consistent with a typical ganglion cyst, and no atypical features were observed.

**Figure 2:**
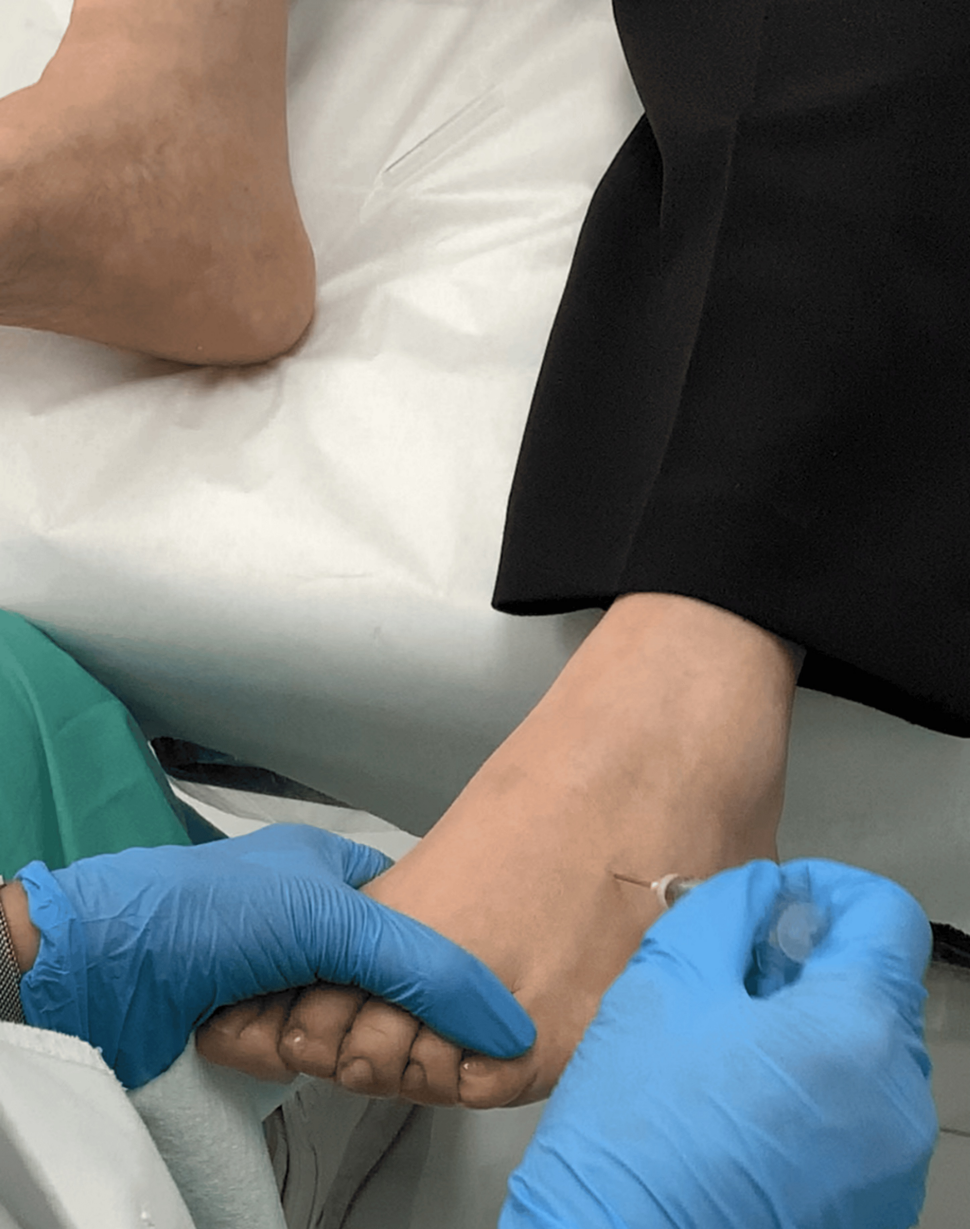
Clinical photograph of the dorsal-lateral aspect of the left midfoot at the base of the fourth metatarsal, depicting the site of the resolved ganglion cyst following aspiration.

Due to the painful nature of the ganglion cyst, aspiration of the lesion was performed, yielding approximately 1 cc of gelatinous, yellow-tinged fluid. Following aspiration, the lesion resolved; therefore, the photograph included depicts the site of the original mass rather than the lesion itself (Figure 2).

**Figure 3:**
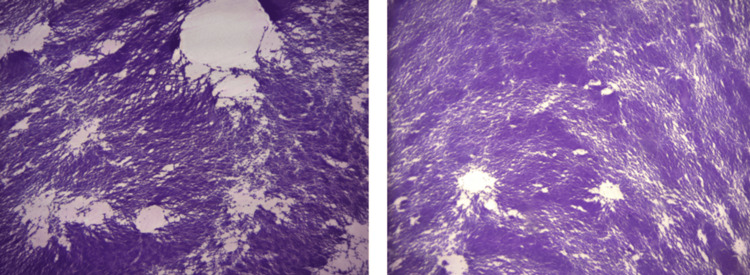
Histopathologic examination of the aspirated material demonstrating scattered mononuclear cells within a proteinaceous background, without acute inflammatory infiltrates, consistent with ganglion cyst contents.

The aspirate was submitted for histopathological examination, which showed few mononuclear cells in a proteinaceous background and no acute inflammatory cells, findings consistent with ganglion cyst contents (Figure 3).

**Figure 4:**
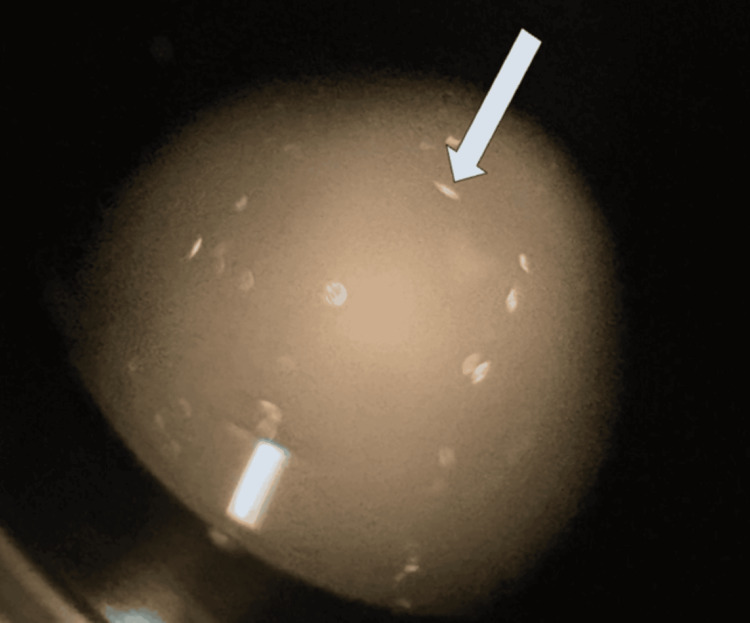
Polarized light microscopy of the aspirated material revealing a needle-shaped crystal (arrow), consistent with urate crystal deposition. The identification of this crystal confirmed localized urate deposition within the ganglion cyst—an atypical finding in the absence of gout or systemic hyperuricemia.

Under polarized light, needle-shaped crystals were present, suggestive of urate crystals (Figure 4).

Subsequent laboratory evaluation of the patient revealed a serum uric acid level of 6.1 mg/dL, at the upper end of normal. Upon additional history-taking, the patient reported that, three months after starting exemestane therapy, she developed mild but persistent arthralgias, described as constant aching in the small joints of the hands and knees, without associated swelling or erythema. She had no prior history of gout or acute gout-like attacks.

## Discussion

Ganglion cysts are common benign soft-tissue lesions. While 60-70% of ganglion cysts arise on the dorsal aspect of the wrist, they may develop in any joint [6]. In the foot, ganglion cysts are typically located around the ankle joint and the dorsum of the foot, as in our case [7].

The aspirated fluid from a typical ganglion cyst is viscous, clear, and rich in hyaluronic acid and mucopolysaccharides [8]. In our patient, however, the aspirated material appeared yellow rather than clear, which prompted histopathologic evaluation. Microscopic examination not only confirmed features consistent with a ganglion cyst but also revealed needle-shaped uric acid crystals under polarized light, an unusual finding not previously reported in the literature. This observation expands the known spectrum of ganglion cyst contents and raises questions regarding possible metabolic or microenvironmental factors that may facilitate crystal deposition in atypical sites.

Uric acid deposition most commonly occurs in the context of gout, an inflammatory arthropathy characterized by monosodium urate crystal accumulation in joints and periarticular soft tissues [9]. In this case, however, there was no clinical history of gout, no acute inflammatory cells present on histology, and only a borderline-elevated serum uric acid level. These findings suggest that the urate crystals were not part of a classic gouty process but rather an incidental or localized deposition. It is possible that the cystic microenvironment, with its high concentration of mucopolysaccharides and hyaluronic acid, contributed to local supersaturation of urate or provided a scaffold that facilitated nucleation of monosodium urate crystals. Such polymers can alter local osmotic balance, bind cations, and create microdomains of reduced solubility, conditions that may promote crystal precipitation even in the absence of systemic hyperuricemia [10].

Another point of interest is the patient’s history of long-term aromatase inhibitor therapy with exemestane for breast cancer. Aromatase inhibitors are well known to cause musculoskeletal symptoms, particularly arthralgias, though no direct link to altered uric acid metabolism has been established [11]. While a causal relationship cannot be confirmed in this case, medication-related changes in periarticular tissue biology could theoretically contribute to a predisposition for crystal deposition. This potential association highlights the importance of considering a patient’s broader clinical context when evaluating unusual findings.

Clinically, this case emphasizes the value of histopathologic examination when the aspirated contents of a ganglion cyst appear atypical. While ganglion cysts are often managed conservatively or diagnosed clinically, aspiration and subsequent microscopic analysis may reveal unexpected features with diagnostic or research significance. The identification of uric acid crystals within this cyst highlights the heterogeneity of these lesions and suggests that routine fluid analysis may be warranted in select cases, particularly when the aspirated fluid is unusual in color, consistency, or associated with patient symptoms.

## Conclusions

This case highlights an unusual presentation of a common lesion, with uric acid crystal deposition identified within a ganglion cyst. Recognition of such atypical findings reinforces the importance of submitting aspirated material for histopathologic analysis when cyst contents appear abnormal in color or consistency. From a clinical standpoint, this approach may help uncover unrecognized metabolic or medication-related contributors, guiding individualized management and follow-up.

Future research could include biochemical analyses of cyst fluid to characterize its composition and crystal-forming potential, in vitro studies exploring the physicochemical conditions that promote urate crystal formation within mucopolysaccharide-rich environments, and clinical investigations evaluating whether aromatase inhibitor therapy, such as exemestane, influences local tissue metabolism or predisposes to crystal deposition. Such studies would help clarify the underlying mechanisms and clinical relevance of this rare association.
